# 5‐Aminolevulinic acid and sodium ferrous citrate ameliorate muscle aging and extend healthspan in *Drosophila*

**DOI:** 10.1002/2211-5463.13338

**Published:** 2021-12-12

**Authors:** Naoko Nozawa, Marie Noguchi, Kanako Shinno, Maki Tajima, Shingo Aizawa, Taro Saito, Akiko Asada, Takuya Ishii, Masahiro Ishizuka, Koichi M. Iijima, Kanae Ando

**Affiliations:** ^1^ Graduate School of Science Tokyo Metropolitan University Japan; ^2^ Division of Pharmaceutical Research SBI Pharmaceuticals Co., Ltd. Tokyo Japan; ^3^ Department of Biological Sciences School of Science Tokyo Metropolitan University Japan; ^4^ Department of Neurogenetics National Center for Geriatrics and Gerontology Obu Japan; ^5^ Department of Experimental Gerontology Graduate School of Pharmaceutical Sciences Nagoya City University Japan

**Keywords:** 5‐aminolevulinic acid, aging, *Drosophila*, mitochondria, muscle architecture, reactive oxygen species

## Abstract

Declines in mitochondrial functions are associated with aging. The combination of 5‐aminolevulinic acid (5‐ALA) and sodium ferrous citrate (SFC) improves mitochondrial functions in cultured cells. In this study, we investigated the effects of dietary supplementation with 5‐ALA and SFC (5‐ALA/SFC) on the healthspan and life span of *Drosophila* *melanogaster*. Adult *Drosophila* fruit flies were fed cornmeal food containing various concentrations of 5‐ALA/SFC. Locomotor functions, life span, muscle architecture, and age‐associated changes in mitochondrial function were analyzed. We found that feeding 5‐ALA/SFC mitigated age‐associated declines in locomotor functions and extended organismal life span. Moreover, 5‐ALA/SFC preserved muscle architecture and maintained the mitochondrial membrane potential in aged animals. Since 5‐ALA phosphate/SFC is used as a human dietary supplement, our results suggest that it could be used to slow the age‐related declines in muscle functions, prevent age‐associated clinical conditions such as frailty, and extend healthspan and life span.

## Graphical abstract

Age‐related declines in physiological systems cause problematic features such as frailty. In this study, we found that 5‐aminolevulinic acid hydrochloride and sodium ferrous citrate (5‐ALA/SFC) mitigated age‐associated declines in locomotor functions and the muscle architecture, preserved the mitochondrial membrane potential, and extended life span in *Drosophila* *melanogaster*. 5‐ALA/SFC might be used to prevent age‐associated clinical conditions.

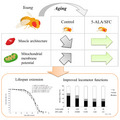

**Abbreviations**
4‐HNE: 4‐hydroxynonenal
5‐ALA: 5‐aminolevulinic acid
ROS: reactive oxygen species
SFC: sodium ferrous citrate

Aging can be defined as the progressive deterioration of the functional properties of cells, tissues, and organs over a life span [1]. Cumulative declines in multiple physiological systems perturb homeostasis and adaptability to internal and external stresses, resulting in increased vulnerability to disease and mortality [2]. Among the problematic features of aging, frailty is a common clinical syndrome characterized by increased vulnerability to disease and mortality due to a decline in the functions of physiological systems. The ability to cope with daily and acute stressors is compromised, which increases the risk of poor health outcomes such as falls, injuries, and mortality [3]. The physical aspects of the frailty phenotype include low grip strength, gait speed, and muscle mass, which overlap with the symptoms of sarcopenia [4]. Although fragility is of increasing concern as populations age, therapeutic interventions for frailty and other age‐related conditions are limited.

The age‐associated physiological decline is linked with mitochondrial dysfunctions [1, 5, 6]. In aged tissues, the activity of mitochondrial enzymes is reduced, respiratory capacity is lower, and there is increased production of reactive oxygen species (ROS). A reduction in mitochondrial function is particularly prominent in highly oxidative tissues such as skeletal muscle [7].

Mitochondrial respiratory chain complexes II, III, and IV, and cytochrome c contain heme; heme deficiency may be an underlying cause of age‐associated mitochondrial dysfunction [8]. The biosynthesis of heme, which is a porphyrin ring complexed with ferrous iron and protoporphyrin IX, starts in mitochondria with the condensation of succinyl‐CoA with the amino acid glycine to generate 5‐aminolevulinic acid (5‐ALA). This process is mediated and rate‐limited by ALA synthase [9]. Heme synthesis, ALA synthase expression, and 5‐ALA levels decline with increasing age [10, 11]. Treatment of cultured mouse cells and human fibroblasts with 5‐ALA and sodium ferrous citrate (SFC) increases the activity of cytochrome c oxidase, the expression of oxidative phosphorylation complexes III, IV, and V, the oxygen consumption rate, and the production of ATP [12, 13, 14]. Thus, we hypothesized that 5‐ALA treatment would protect muscle function by increasing mitochondrial ATP synthesis during aging.

In this study, we investigated the effects of 5‐ALA/SFC on age‐related declines in physical activity and life span using the fruit fly *Drosophila melanogaster*. *Drosophila* is a genetically tractable model system with the relatively short life span and is used to study human diseases and aging. Age‐associated dysfunctions are prominently observed in the muscle [15]. Here, we report that supplementation of food with 5‐ALA/SFC significantly mitigated age‐associated locomotor decline, extended the organismal life span, improved sarcomere structure, and maintained the mitochondrial membrane potential in aged muscle tissue.

## Methods

### Fly stocks and husbandry

Adult flies (*w* ^1118^) were maintained in standard cornmeal media (10% glucose, 0.7% agar, 9% cornmeal, 4% yeast extract, 0.3% propionic acid, and 0.1% *n*‐butyl *p*‐hydroxybenzoate) containing 5‐ALA/SFC at 25 °C under light–dark cycles of 12 : 12 h. The flies were transferred to fresh food vials for every 2–3 days.

### 5‐ALA/SFC feeding

Flies were raised on the regular cornmeal. After eclosion, male flies were maintained on regular cornmeal food mixed with 5‐ALA/SFC at the indicated concentration. Flies were placed at 30 flies/vial, and food vials were changed every 2–3 days.

### Chemicals

5‐ALA hydrochloride (neo ALA Co. Ltd, Tokyo, Japan) and SFC (Komatsuya Corporation, Osaka, Japan) were provided by SBI Pharmaceuticals Co., Ltd. (Tokyo, Japan) Phalloidin (Sigma‐Aldrich, St. Louis, MO, USA), TOPRO‐3 (Invitrogen, Waltham, MA, USA), anti‐ATP5A (Abcam, Cambridge, UK), MitoTracker Deep Red FM (Thermo Fisher, Waltham, MA, USA), MT‐1 MitoMP Detection Kit (DOJINDO LABORATORIES, Kumamoto, Japan), anti‐4‐hydroxynonenal (4‐HNE; Abcam), Alexa Fluor 488 anti‐mouse IgG (Thermo Fisher), Alexa Fluor 488 anti‐rabbit IgG (Thermo Fisher), sucrose, Tris, MgCl_2_, aminohexanoic acid, Bis‐Tris, Coomassie Blue G (FUJIFILM Wako Pure Chemical, Osaka, Japan), Triton X‐100 (Sigma), anti‐citrate synthase (CISY11‐A; Alpha Diagnostics, San Antonio, TX, USA), anti‐rabbit IgG HRP‐Linked Whole Ab Donkey (GE Healthcare, Chicago, IL, USA), and ImmunoStar LD (FUJIFILM Wako Pure Chemical) were purchased.

### Quantification of 5‐ALA levels in the fly food

5‐ALA levels were analyzed as previously described with a modification [16]. Briefly, fly food was mixed with 8 times volume of water and centrifuged (2655 *g*, 10 min at 4 °C). The supernatant was collected and the process was repeated, and subjected to fluorometric analysis as described in Ref. [16].

### Climbing assay

The climbing assay was performed as previously described [17]. Approximately 30 flies were placed in an empty plastic vial (2.5 cm in diameter × 8 cm in length). The vial was gently tapped to knock the flies to the bottom, and the height that the flies climbed in 10 s after tapping to the bottom of the vials was measured. Experiments were repeated more than three times, and a representative result was shown. Food vials were changed every 2–3 days.

### Life span analysis

Food vials containing approximately 25 flies were placed on their sides at 25 °C under conditions of a 12‐h : 12‐h light : dark cycle. Food vials were changed every 2–3 days, and the number of dead flies was counted each time. At least three vials for each genotype were prepared.

### Phalloidin staining

The thoracic muscles of male flies were dissected in cold Schneider's *Drosophila* medium (SDM; Thermo Fisher Scientific). First, an incision was made in the middle of the thorax, and then, indirect flight muscles were dissected from the thorax. The muscles were then fixed with 4% paraformaldehyde (Wako) for 30 min at room temperature, incubated with 2 µg·mL^−1^ phalloidin/tetramethylrhodamine B isothiocyanate peptide (Sigma‐Aldrich) in PBS overnight on a shaker at 4 °C, and imaged under a laser confocal microscope (Zeiss LSM 710, Oberkochen, Germany) and analyzed with imagej [18].

### Blue NativePAGE

Blue NativePAGE to analyze mitochondrial respiratory chain protein was carried out as described with a modification [19, 20]. Thorax from 35 flies was homogenized in 1 ml of chilled mitochondrial isolation medium (250 mm sucrose, 10 mm Tris/HCl (pH 7.4), 0.15 mm MgCl_2_). The samples were centrifuged twice for 10 min at 600 *g* at 4 °C to remove debris. The supernatant was centrifuged again for 10 min at 7000 *g* at 4 °C. For BN‐PAGE analyses, the NativePAGE Novex Bis‐Tris Gel System (Life Technologies, Carlsbad, CA, USA) was used according to the manufacturer's protocol. Mitochondrial fractions were solubilized in sample buffer (50 mm NaCl, Tris‐HCl (pH 7.4) and 1% Triton X‐100). After centrifugation for 5 min at 17,800 *g* at 4 °C, the supernatants were collected. Mitochondrial protein levels were determined using a BCA assay. 30 µg mitochondrial protein was mixed with 10× loading dye solution (5% Coomassie Blue G, 1 m aminohexanoic acid, 100 mm Bis‐Tris) and separated on 3%–12% NativePAGE gels. Gels were stained with Coomassie Blue.

### Western Blot

10 µg of mitochondrial protein was separated by 4–15% polyacrylamide gel electrophoresis, transferred to polyvinylidene difluoride membranes using the Trans‐Blot® Turbo Transfer System (Bio‐Rad Laboratories, Hercules, CA, USA), and incubated with antibodies. Immunolabeled proteins were detected using a chemiluminescence kit (ImmunoStar LD) and a lumino‐image analyzer [ChemiDoc MP System (Bio‐Rad Laboratories)]. The primary antibodies used were rabbit anti‐citrate synthase. The secondary antibodies used were anti‐rabbit IgG HRP‐Linked Whole Ab Donkey (GE Healthcare).

### Citrate synthase activity assay

Thoraxes from the 20 flies were homogenized, and citrate synthase activity was measured by using Citrate Synthase Activity Colorimetric Assay Kit (BioVision, Milpitas, CA, USA) according to the manufacturer's manual. Protein levels were measured with the BCA Protein Assay Kit (Thermo Fisher Scientific).

### Bilirubin assay

Twenty thoraxes were homogenized, and bilirubin levels were measured by using Bilirubin Assay Kit (Cell Biolabs, Inc., San Diego, CA, USA) according to the manufacturer's instruction.

### qRT‐PCR

Total RNA of the 20 thoraxes was purified according to the protocol provided with the RNeasy Plus Universal Mini Kit (Qiagen, Hilden, Germany). The amount and purity of the total RNA were measured using the NanoDrop One Spectrophotometer (Thermo Scientific). cDNA was synthesized from 0.5 μg of total RNA using the High Capacity RNA‐to‐cDNA Kit (Life Technologies). The expression level of each gene was measured using the PowerUp SYBR Green Master Mix (Life Technologies) and the StepOnePlus Real‐Time PCR System (Life Technologies). Expression of genes of interest was standardized relative to *rp49*. Primers were designed using DRSC FlyPrimerBank (Harvard Medical School, Boston, MA, USA). Primer sequences are shown in Table S1.

### Analysis of mitochondrial morphology

Male flies were anesthetized, and the indirect flight muscle was dissected in cold SDM. The muscles were then fixed for 30 min with 4% paraformaldehyde at room temperature. After fixation, samples were washed three times with 0.1% PBST (0.1% triton in 1× PBS) for 5 min each time. The samples were blocked with 1% normal goat serum (NGS)/0.1% PBST for 30 min at room temperature. The samples were then incubated with anti‐ATP5A antibody (1 : 250; Abcam) in 1% NGS/0.1% PBST overnight at 4 °C. The next day, the samples were washed three times for 5 min in 0.1% PBST and then incubated in 1% NGS with 0.1% PBST containing Alexa Fluor 488 anti‐mouse IgG (Thermo Fisher) at room temperature for 3 h. Subsequent to this, the samples were washed three times in 0.1% PBST and mounted in VectaShield mounting medium (Vector Laboratories, Burlingame, CA, USA). Images were captured using a laser confocal microscope (Zeiss LSM 710) and analyzed with ImageJ [18].

### Mitochondrial membrane potential assay

Male flies were anesthetized, and the indirect flight muscles were dissected in cold SDM. The muscles were then incubated in SDM containing 100 nm MitoTracker Deep Red FM (Thermo Fisher Scientific) and MT‐1 Dye (1 : 1000; DOJINDO LABORATORIES) for 30 min at room temperature. Samples were washed three times for 5 min each time with SDM, and fixed with 4% paraformaldehyde for 40 min at room temperature. After fixation, the samples were washed three times for 5 min each time with SDM. The samples were imaged under a laser confocal microscope (Zeiss LSM 710) and analyzed with ImageJ [18].

### 4‐HNE staining

Male flies were anesthetized, and the indirect flight muscles were dissected in cold SDM. The muscles were then fixed for 40 min with 4% paraformaldehyde at room temperature. After fixation, samples were washed three times with 0.1% PBST (0.1% triton in 1× PBS) for 5 min each time. The samples were blocked with 1% NGS/0.1% PBST for 5 h at room temperature. The samples were then incubated with anti‐4‐HNE antibody (1 : 100; Abcam) in 1% NGS/0.1% PBST overnight at 4 °C. The next day, the samples were washed three times for 15 min in 0.1% PBST. Alexa Fluor 488 anti‐rabbit IgG (Thermo Fisher) and phalloidin were then added to the samples, and kept at room temperature for 3 h. Subsequent to this, the samples were washed three times in 0.1% PBST and mounted in VectaShield mounting medium (Vector Laboratories). Images were captured using a laser confocal microscope (Zeiss LSM 710) and analyzed with imagej [18].

### Statistics

Statistics were done with Microsoft Excel (Microsoft, Redmond, WA, USA), Statcel‐the Useful Addin Forms on Excel‐3rd ed. (OMS Publishing, Tokyo, Japan), Proc Lifetest of sas version 9.4 (SAS, Cary, NC, USA), graphpad prism9 (GraphPad Software, MDF Co. Ltd, Tokyo, Japan), and r (R Core Team, 2021) [21]. Differences were assessed using one‐way ANOVA or logrank test followed by Dunnett's test or Tukey's HSD. *P* values < 0.05 were considered statistically significant.

## Results

### 5‐ALA is stable in fly food for up to 2 weeks

To examine the effect of 5‐ALA on aging and life span, we maintained flies on a diet of cornmeal food containing 5‐ALA and SFC. The addition of 5‐ALA (100 µm or higher) and SFC to the culture medium of cells significantly increases the cellular oxygen consumption rate [12]. However, it is not clear whether 5‐ALA/SFC mixed with food is efficiently absorbed and distributed to fly tissues. Since the concentration of dietary chemicals used in most fly studies is higher than the concentrations of chemicals used in cultured cells [22, 23, 24, 25, 26], we prepared food containing 0.05, 0.5, 5, and 50 mm 5‐ALA hydrochloride combined with SFC at a ratio of 20 : 1.

We analyzed the stability of 5‐ALA in the food media with or without flies. 5‐ALA was stable for at least two weeks in fly food stored at 25 or 4 °C (Table 1). Moreover, the 5‐ALA concentration did not reduce when flies were raised in food (Table 2), indicating that the presence of fly excrement does not affect the 5‐ALA concentration in food.

**Table 1:** 5‐ALA is stable in fly food for up to 2 weeks. Fly food was mixed with 50 mm 5‐ALA and 2.5 mm SFC. The concentration of 5‐ALA was analyzed on the day on which the food was prepared (day 0) and after it had been stored for 1 week (day 7) or 2 weeks (day 14) at 4 or 25 °C. The concentration of 5‐ALA in food stored at 25 °C was higher due to water evaporation (see the column headed ‘relative weight of food’). The expected concentrations of 5‐ALA without water evaporation are shown in brackets.

	Amount of 5‐ALA in 100 g of food (g)				Relative weight of food (%)			5‐ALA concentration (mm)			
	Theoretic al value	Day 0	Day 7	Day 14	Day 0	Day 7	Day 14	Theoretical value	Day 0	Day 7	Day 14
4 °C	0.60	0.54	0.54	0.53	100	99	98	50	44.5	44.5	44.0
25 °C			0.70	0.88		77	58			58.2 (49.2)	73.0 (50.8)

**Table 2:** 5‐ALA is stable in food containing cultured flies. Flies were cultured in food containing 5‐ALA at 25 °C. When next‐generation flies eclosed after 10 days (day 10), flies and larvae were removed from the food, and the 5‐ALA concentration was analyzed.

	Amount of 5‐ALA in 100 g of food (g)		5‐ALA concentration (mm)	
	Day 0	Day 10	Day 0	Day 10
25 °C with fly	0.52	0.66	52.5	67.5

### 5‐ALA/SFC ameliorates the age‐related decline in locomotor activity

**Fig. 1:**
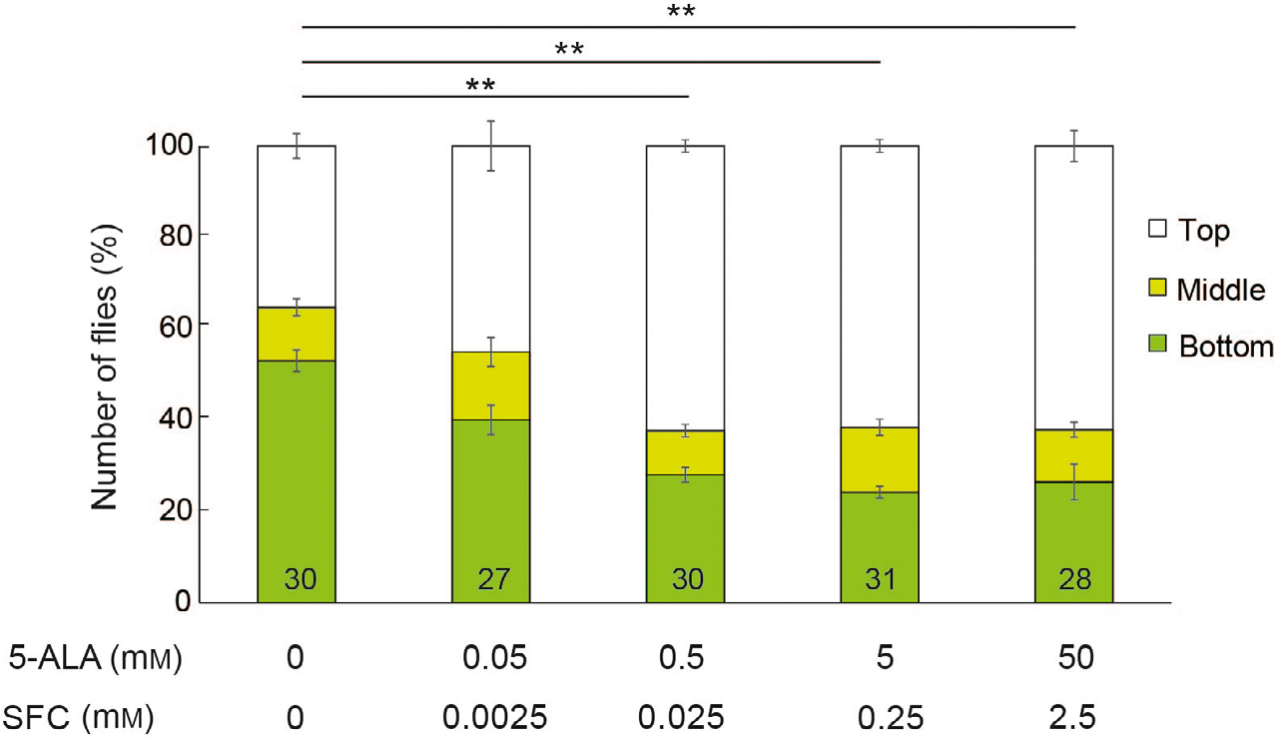
5‐ALA/SFC ameliorates the age‐associated decline in the locomotor activity of flies. Flies (22 days old) were tapped to the bottom of a vial, and the percentages of flies located at the top, middle, and bottom of the vial after 10 s were calculated. Data are expressed as mean ± SE. Numbers in the bars indicate sample size (number of flies); **P < 0.01; one‐way ANOVA followed by Dunnett's test.

We used a climbing assay [27], which is a robust behavioral assay that takes advantage of innate negative geotaxis behavior, to analyze the effects of 5‐ALA/SFC on the age‐related declines in locomotor function. Flies were raised in regular cornmeal food and after eclosion were maintained in cornmeal food containing various concentrations of 5‐ALA and SFC. Flies maintained on regular food begin to show an age‐dependent decline in climbing ability around 3 weeks of age [27]. Supplementation of the diet with 5‐ALA/SFC significantly improved the locomotor activity of 3‐week‐old flies in a dose‐dependent manner (Fig. 1). Locomotor function was significantly improved by 0.05 mm 5‐ALA and 0.0025 mm SFC and was further improved by higher concentrations (Fig. 1). These results suggest that dietary supplementation with 5‐ALA/SFC counteracts an age‐related physiological decline.

### 5‐ALA/SFC extends life span

**Fig. 2:**
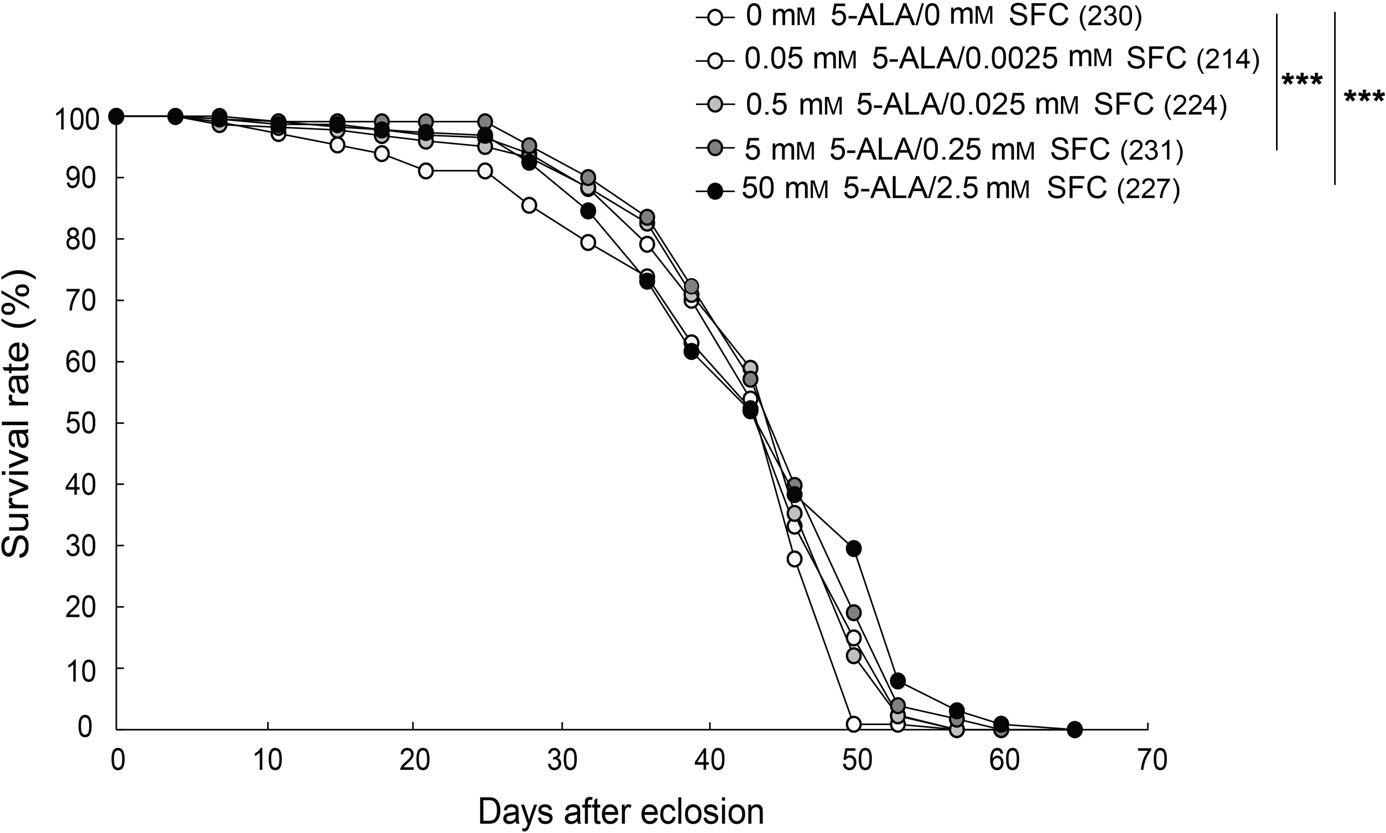
5‐ALA/SFC extends life span. Flies were raised on regular cornmeal food then maintained on food containing the indicated concentrations of 5‐ALA and SFC after eclosion. The percentage of surviving flies was determined on various days (indicated on the *x* = axis) after eclosion. Numbers in parentheses indicate sample size (the number of flies). ***P < 0.005, logrank test.

Next, we analyzed the effect of dietary 5‐ALA/SFC supplementation on life span. The life span of flies maintained on food containing 5 mm 5‐ALA and 0.25 mm SFC or 50 mm 5‐ALA and 2.5 mm SFC was significantly extended (*P* < 0.001, *n* = 214–231, logrank test). The maximum life span of control flies was 57 days, while the maximum life span of flies fed food containing 5 mm 5‐ALA and 0.25 mm SFC was 60 days, and the maximum life span of flies fed food containing 50 mm 5‐ALA and 2.5 mm SFC was 65 days (Fig. 2). These results indicate that 0.5 mm 5‐ALA/0.025 mm SFC extends healthspan, and 5 mm 5‐ALA/0.25 mm SFC or higher concentrations extend both healthspan and life span.

### 5‐ALA/SFC maintains muscle architecture during aging

**Fig. 3:**
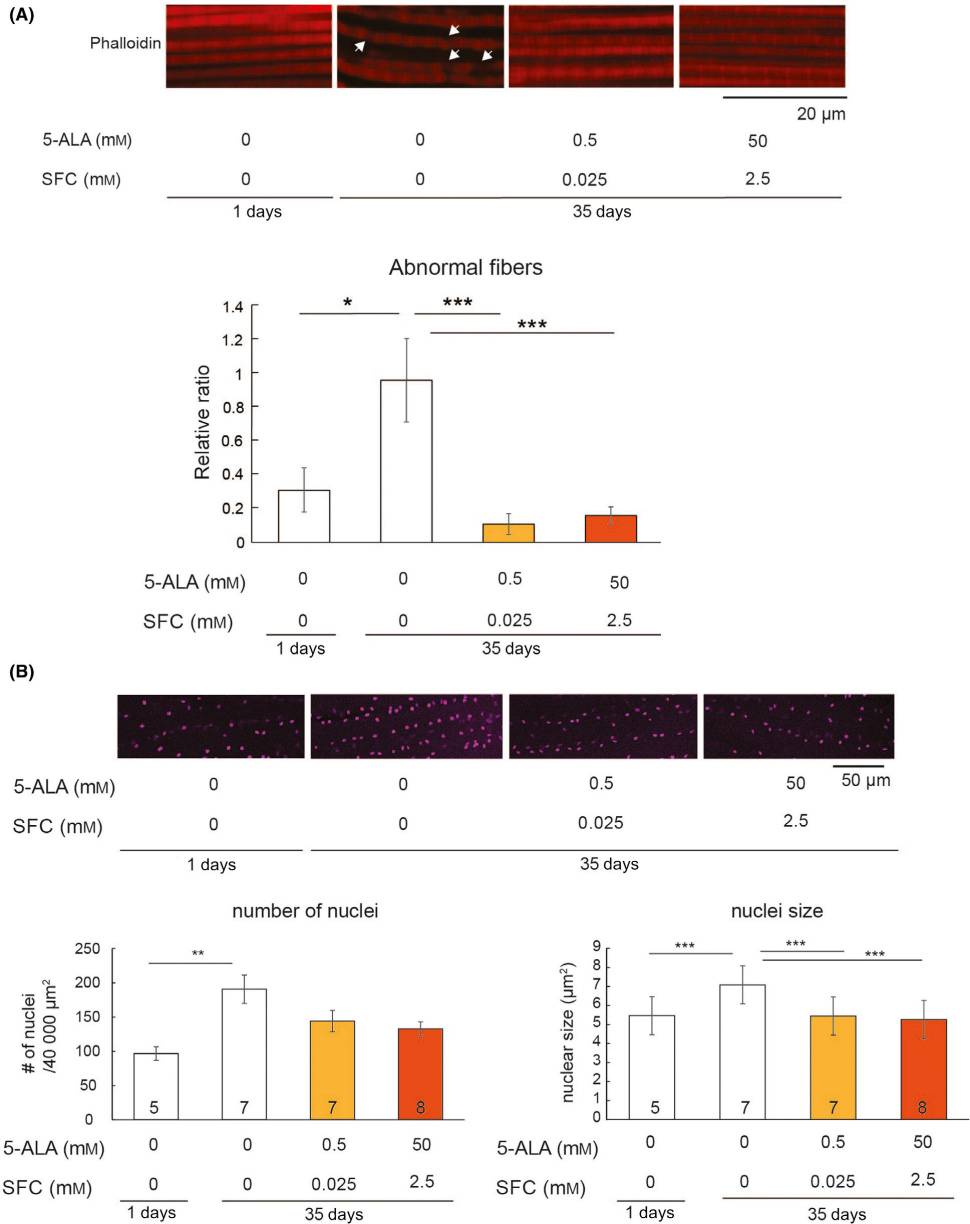
5‐ALA/SFC maintains muscle integrity. (A) Indirect flight muscles were dissected from young (1 day old) or aged (35 days old) flies and stained with phalloidin (red) to detect sarcomeres. The disruption of sarcomere structures in aged muscles is indicated by the arrows. Representative images are shown in the top panel, and quantitation of the data is shown in the bottom panel. Data are expressed as mean ± SE, *n* = 6; *P < 0.05, ***P < 0.005; one‐way ANOVA followed by Tukey's test. Scale bar = 20 µm. (B) Nuclei in the indirect flight muscles were stained with TOPRO‐3 (magenta). Representative images are shown in the top panel, and quantitation of the data is shown in the bottom panel. (Left) Number of nuclei; (Right) the size of nuclei. Numbers in the bars indicate the sample size (the number of muscle samples). Data are expressed as mean ± SE; **P < 0.01, ***P < 0.005; one‐way ANOVA followed by Tukey's test. Scale bar = 50 µm.

Muscle integrity is essential for locomotor function and declines during aging [28]. We analyzed the sarcomere structure in the indirect flight muscles of aged flies treated with or without 5‐ALA/SFC. Muscle architecture was disordered in control flies by 35 days of age. Sarcomere structures were disrupted in aged flies (arrows), but not in young flies (Fig. 3A, compare 0 mm 5‐ALA/0 mm SFC at 1 and 35 days). In flies fed food containing 5‐ALA/SFC, sarcomeres were more clearly observed (Fig. 3A, *P *< 0.005, compare 0 mm 5‐ALA/0 mm SFC and 0.5 mm 5‐ALA/0.025 mm SFC or 50 mm 5‐ALA/2.5 mm SFC in 35‐day‐old flies).

The number, size, and arrangement of myonuclei are associated with muscle function [29]. Myonuclei in the center of the myofiber are called central nuclei; they are associated with myofibers that have degenerated and regenerated, and are a prominent feature of aged skeletal muscle in mice [11, 12, 13, 14, 15, 16]. We found that the density of myonuclei was greater in aged flies than in young flies (Fig. 3B, compare 1‐day‐old flies 0 mm 5‐ALA/0 mm SFC and 35‐day‐old flies 0 mm 5‐ALA/0 mm SFC, *P* < 0.006). By contrast, there was no significant difference in myonuclear density between young and aged flies supplemented with 5‐ALA/SFC (*P* > 0.05 between 1‐day‐old flies 0 mm 5‐ALA/0 mm SFC and 35‐day‐old flies 0.5 mm 5‐ALA/0.025 mm SFC, or between 1‐day‐old flies 0 mm 5‐ALA/0 mm SFC and 35‐day‐old flies 50 mm 5‐ALA/2.5 mm SFC).

These results suggest that the muscle of aged flies treated with 5‐ALA/SFC maintains a key feature, namely myonuclear density, which is usually associated with young muscle.

### 5‐ALA/SFC maintains mitochondrial membrane potential

**Fig. 4:**
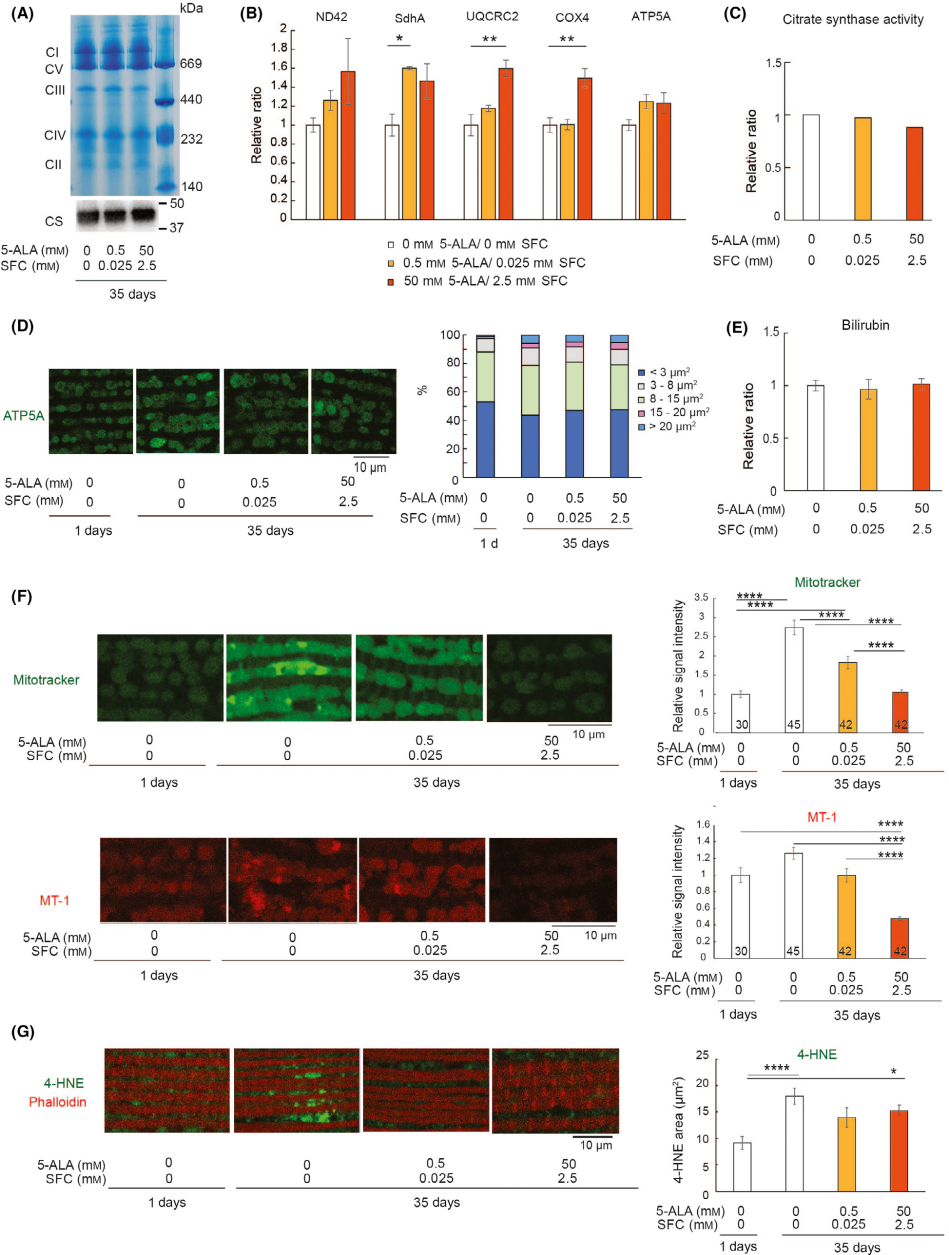
5‐ALA/SFC maintains the mitochondrial membrane potential in the aged muscle. (A) (Top panel) Blue NativePAGE of thorax from male flies at 35 days old. (CI, complex I; CV, complex V; CIII, complex III; CIV, complex IV; CII, complex II). (Bottom panel) Western blot analysis of the samples with an anti‐citrate synthase antibody. *n* = 3 and a representative image is shown. (B) 5‐ALA/SFC slightly increased the levels of mRNA of mitochondrial enzymes. qRT‐PCR from the thorax of flies at 35 days old. Data are expressed as mean ± SE, *n* = 3; *P < 0.05, **P < 0.01; one‐way ANOVA followed by Dunnett's test. (C) Treatment with 5‐ALA/SFC did not alter citrate synthase activity. Citrate synthase activity was normalized to protein levels and shown as the ratio relative to flies not treated with 5‐ALA/SFC. The data shown are from an average of two independent experiments. (D) 5‐ALA/SFC did not affect mitochondrial sizes significantly. Indirect flight muscles were dissected from young (1 day old) or aged (35 days old) flies and stained with an anti‐ATP5A antibody (green) to detect mitochondria. Representative images are shown on the left, and quantitation of the data is shown on the right. Data are expressed as mean, *n* = 3; *P* > 0.05; chi‐squared test. Scale bar = 10 µm. (E) Treatment with 5‐ALA/SFC did not alter bilirubin levels. Bilirubin levels were normalized with protein levels and expressed as the ratio relative to the flies not treated with 5‐ALA/SFC treatment. Data are expressed as mean ± SE, *n* = 3; *P* > 0.05; one‐way ANOVA followed by Dunnett's test. (F) Indirect flight muscles were stained with the mitochondrial dye MitoTracker Deep Red FM (green) and MT‐1 (red). Representative images (left) and quantification of the signal intensity (right) are shown. Data are expressed as mean ± SE, and numbers in the bars indicate *n*; ****P < 0.0001; one‐way ANOVA followed by Tukey's test. Scale bar = 10 µm. (G) The indirect flight muscles were stained with an anti‐4‐HNE antibody (green) and phalloidin (red). Representative images (left) and quantification of the areas of 4‐HNE aggregates in the muscle (right) are shown. Data are expressed as mean ± SE, *n* = 25; *P < 0.05, ****P < 0.0001; one‐way ANOVA followed by Tukey's test. Scale bar = 10 µm.

Mitochondrial dysfunctions occur in aged muscle tissues [15]. We sought to determine whether the protective effects of 5‐ALA/SFC are accompanied by improved mitochondrial functions. Analyses of the expression of mitochondrial respiratory complexes by blue NativePAGE showed that the aged flies with or without 5‐ALA/SFC expressed similar levels of mitochondrial respiratory complexes (Fig. 4A). The levels of mRNA coding respiratory chain proteins such as ND42, SdhA, UQCRC2, COX4, and ATP5A were further analyzed by qRT‐PCR. 5‐ALA/SFC increased the levels of mRNA coding SdhA, UQCRC2, and COX4 but not the levels of ND42 or ATP5A (Fig. 4B). The levels and activity of citrate synthase were similar with or without 5‐ALA/SFC treatment (Fig. 4A,C), suggesting that 5‐ALA/SFC does not alter mitochondrial activity. Mitochondrial dynamics, such as fission and fusions, act as quality control mechanisms and decline during aging [30]. We analyzed mitochondrial morphology by immunostaining with anti‐ATP5A and found that 5‐ALA/SFC feeding did not affect mitochondrial size in the indirect flight muscle in aged flies (Fig. 4D). We also investigated whether 5‐ALA/SFC affects overall heme metabolism in flies. Treatment with 5‐ALA/SFC feeding did not significantly change the levels of bilirubin, the catabolic product of heme metabolism (Fig. 4E).

Finally, we analyzed the mitochondrial membrane potential by staining dissected tissues with mitochondrial membrane potential‐sensitive dyes. We used MitoTracker, whose accumulation in mitochondria is dependent upon their membrane potential and preserved after fixation. We found that the MitoTracker signal intensity was significantly increased in the muscles in aged flies and was reduced by 5‐ALA/SFC (Fig. 4F, compare 0 mm 5‐ALA/0 mm SFC and 0.5 mm 5‐ALA/0.025 mm SFC or 50 mm 5‐ALA/2.5 mm SFC). We also used MT‐1, another cationic dye sequestered to mitochondria by their membrane potential [31]. MT‐1 signals in young animals and those in aged animals were not significantly different, while 5‐ALA/SFC significantly reduced them (Fig. 4F, compare 0 mm 5‐ALA/0 mm SFC and 50 mm 5‐ALA/2.5 mm SFC).

The rate of ROS production depends on mitochondrial membrane potential, and hyperpolarization of mitochondria could lead to an increase in ROS production [32]. 5‐ALA/SFC may preserve the mild depolarization of the mitochondrial inner membrane to reduce ROS production. To test this hypothesis, we analyzed ROS levels by using 4‐HNE, a product of lipid peroxidation and a biomarker of oxidative stress [33]. The aged flies exhibited significantly increased 4‐HNE staining, indicating increased oxidative damage (Fig. 4G, compare 1 and 35 days with 0 mm 5‐ALA/0 mm SFC, *P* < 0.001). By contrast, no or less significant differences in 4‐HNE signal were found between young flies and aged flies on the diet containing 5‐ALA/SFC (compare 1‐day‐old 0 mm 5‐ALA/0 mm SFC and 35‐day‐old 0.5 mm 5‐ALA/0.025 mm SFC, or 1‐day‐old 0 mm 5‐ALA/0 mm SFC and 35‐day‐old 50 mm 5‐ALA/2.5 mm SFC). These results suggest that the supplementation of 5‐ALA/SFC counteracts the age‐associated decline in the mechanisms regulating mitochondrial activities.

## Discussion

Administration of 5‐ALA/SFC ameliorates mitochondrial dysfunction in fibroblasts from individuals with mitochondrial diseases [12] and in white adipose tissue from mice with diet‐induced obesity [13]. Treatment with 5‐ALA/SFC also improves muscle function in mice with sarcopenia and in a mouse model of chronic kidney disease [34]. Here, we report for the first time that 5‐ALA/SFC protects against organismal aging. The muscle architecture in aged flies treated with 5‐ALA/SFC was similar to that in young animals (Fig. 3); this effect of 5‐ALA/SFC on muscle architecture may contribute to its protective effects on age‐dependent declines in locomotion (Fig. 1).

Reactive oxygen species induce oxidative damage to cellular macromolecules due to their high chemical reactivity and are believed to be a significant cause of aging [35]. The respiratory chain is the primary production site of superoxide, and ROS are formed as a by‐product of oxidative phosphorylation [35]. The rate of ROS production depends on mitochondrial membrane potential and the activity of the respiratory complexes [32]. Dissipation of the mitochondrial membrane potential could increase ROS generation when respiration is inhibited [36]. On the contrary, hyperpolarization of mitochondria can also lead to ROS production [32, 37]. In fact, mild depolarization of the mitochondrial inner membrane has been reported as a defense mechanism that prevents oxidant‐mediated damage by reducing mitochondrial ROS generation through an ADP‐recycling mechanism [36, 38, 39, 40]. This mild depolarization decreases during aging in the skeletal muscle of mice [41], suggesting that elevated oxidative damages with aging may be partly caused by the loss of mild depolarization of the mitochondrial inner membrane. In agreement with this, we found that 5‐ALA/SFC treatment reduced the mitochondrial membrane potential and oxidative damages in the aged muscles (Fig. 4). These results suggest that 5‐ALA/SFC has a novel mechanism of action that benefits physical functions. Further analyses of the molecular mechanisms by which 5‐ALA/SFC regulates mitochondrial membrane potential would enhance our understanding of the roles of mitochondria in aging.

5‐ALA is a natural delta amino acid widely present in nature [42]. In addition, 5‐ALA/SFC is consumed as a dietary supplement in several countries [43, 44]. Age‐associated conditions such as frailty are caused by cumulative damage over a long time [45], so a dietary supplement might be a suitable format for preventing age‐associated conditions. Our results suggest that 5‐ALA/SFC is a possible therapeutic intervention for preventing frailty in elderly people.

## Conclusions

Oral administration of 5‐ALA/SFC mitigates age‐dependent declines in locomotor function, extends life span, and maintains muscle architecture and mitochondrial membrane potential in aged *Drosophila*. Our results also suggest that 5‐ALA/SFC supports the maintenance of the mitochondrial membrane potential and suppresses ROS generation. Together, our findings suggest that 5‐ALA/SFC may be a possible therapeutic intervention for age‐related declines in muscle functions and for preventing important symptoms of aging such as frailty.

## Conflict of interest

Naoko Nozawa, Takuya Ishii, and Masahiro Ishizuka are employees of SBI Pharmaceuticals Co., Ltd. No other conflict of interest.

## Author contributions

NN, KMI, and KA designed the experiments. NN, MN, KS, MT, AA, TS, SA, and KA performed the experiments. NN and KA analyzed the data. NN, MN, TI, MI, KMI, and KA wrote the manuscript.

## Supporting information


**Table S1**. Primer sequences (5′–3′).

## Data Availability

All data generated or analyzed during this study are included in this published article.
