# Supplementary material for: 5‐Aminolevulinic acid and sodium ferrous citrate ameliorate muscle aging and extend healthspan in Drosophila
Source: FEBS Open Bio. 2021 Dec 12;12(1):295–305. doi: 10.1002/2211-5463.13338 (PMC8727951; doi:10.1002/2211-5463.13338)
Supplement: Supplementary file 1 — Table S1. Primer sequences (5′–3′). [file FEB4-12-295-s001.docx]

**Supplementary Table 1.** Primer Sequences (5′–3′)

| Target Gene | Forward | Reverse |
| --- | --- | --- |
| ND42 | ACACAGTCGCCCAATCTACTC | GCTGTACTTCTTCGTCTTGTACG |
| SdhA | TGTACGACACGGTCAAGGG | TTCTCCAGCTCAATGACAGCC |
| UQCRC2 | AATGCCAAAACGGTGGTCAAC | GGGGCGAGTAGATCGAGTT |
| COX4 | TACGATGAGCTGCCCGTTAC | GGTTGATTTCCAGGTCGATGAT |
| ATP5A | CCGTTTCCGTGTGGGAATCAA | AGAGCGGTCTTACCAGTCTGA |
| Rp49 | GCTAAGCTGTCGCACAAATG | GTTCGATCCGTAACCGATGT |
